# Obituary for Paul Bacon, Emeritus Professor of Rheumatology, University of Birmingham, UK

**DOI:** 10.31138/mjr.29.1.62

**Published:** 2018-03-19

**Authors:** George Kitas

**Affiliations:** Chief Editor

It is with great sadness that we report the death of Paul Bacon, Emeritus Professor of Rheumatology at the University of Birmingham in the UK and one of the very first senior members of this Journal’s Advisory Board. His passing was unexpected for a man that remained extremely active in the international world of rheumatology but also life in general a good 15 years after his retirement as the first Arthritis Research UK Professor of Rheumatology in Birmingham. It followed a short illness and was quite peaceful, with family and close friends around.

I met Professor Bacon in late 1985, when, as a just qualified medical doctor from Greece, I went to Britain to do my PhD in his relatively new department in Birmingham. I was privileged to associate with him until the end, initially as my teacher and trainer, later as my mentor and then, for a quarter of a century, as a colleague, collaborator but mostly close personal and family friend – a person that played an enormous role in my professional and personal life. This makes me sufficiently “qualified” to say a few words about the “human dimension” of Paul and his wife, Jean, which demonstrates a philosophy of life reflected in Paul’s professional achievements. Paul and Jean went out of their way to help young trainees from the UK and elsewhere settle in and feel comfortable in their new environment; took a keen interest in our wellbeing outside professional confines; helped several people in many different ways get through difficult times; and at the same time contributed enormously to our professional development in the UK or abroad. I will not say much more about these human qualities, as I perceive them from my personal experience with him over the years, but will draw on one of them which is of wider importance: Paul was a true internationalist well before “globalisation” became a fashionable word. He believed in people developing “knowhow” anywhere but then disseminating it in their own units, countries, people - for wider benefit. He did this himself with long visits to the USA, Australia and, towards the end, India, always bringing back new ideas which he pursued with great enthusiasm. He cultivated this attitude to others and I could give many examples from the UK and elsewhere – this is why he was such a keen supporter of this Journal and its aims, and it is a great pity that he did not manage to finish a commentary he was writing for the Mediterranean Journal of Rheumatology about his scientific and other experiences in India and the importance of international collaboration between rheumatologists and scientists. He was a firm believer that knowledge exists everywhere, as long as we are receptive, and that collaboration through networking achieves usually more than individual scientific endeavour, at least in the world of Medicine.

Paul Bacon has left a massive legacy in the world of Rheumatology and the overwhelming majority of his achievements were through collaboration and networking. Probably his major contribution was the practical introduction of the concept of “measuring” disease activity in conditions such as vasculitis and Lupus, and of separating the concept of disease activity from that of damage and from quality of life. Indices that are now “common place”, such as the Birmingham Vasculitis Activity Score (BVAS), the Vasculitis Damage Index (VDI), the British Isles Lupus Assessment Group (BILAG) and subsequently the Systemic Lupus International Collaborating Clinics (SLICC) indices were developed with leadership from Paul, collaboration with many of the “big names” of rheumatology and other specialities of the 1980s–1990s era from all over the world, and the very hard work of many people in his department who are now themselves international leaders in their fields. The development and validation of these tools and the respective collaborative networks enabled virtually all of the post-1990s clinical trials in lupus and systemic vasculitis, which have produced the evidence that define contemporary clinical practice internationally. Even after his retirement, Paul was instrumental in the development of ITAS, the Indian Takayasu’s Activity Score, with colleagues from India, where the disease is particularly prevalent.

Well before most others, Paul Bacon saw the enormous value of sub-specialty clinics serving many purposes: the development by rheumatologists of particular expertise in relatively rare conditions and use of this for “skills-transfer” both in the clinical and the research arenas; the promotion of better standards of care for patients; and the facilitation of good quality clinical and basic research by the availability of ample and well-phenotyped clinical material. This was particularly progressive, if one considers that it was at a time that rheumatology had just been born as an independent specialty in the UK. Thus, subspecialty clinics were created, for early arthritis, lupus, vasculitis, scleroderma and even paediatric rheumatology, amongst others. These provided fertile ground for many rheumatologists, clinical and non-clinical scientists, who have formed the next generation of rheumatology academics and clinicians that is now itself making enormous contributions to the specialty. Paul foresaw many other developments too: for example the value of community-based patient education centres and the use of the web (in the 90s!) for patient education and self-assessment. Many of us who were lucky enough to live through this time, used different versions of this same model to develop our own departments, research interests and career paths (whether we like to admit it or not).

In this era of “metrics”, Paul’s contribution to the world of international rheumatology is not his >380 PubMed publications and >20000 citations. Much more important is the human resource he helped develop, including the number of clinical and non-clinical Professors in the UK and abroad (around 20, by far the largest for a single rheumatology department) - they are now themselves leaders, leaving their own stamp on the specialty; it is the even higher number of rheumatologists, mostly in the UK but several also abroad, that have somehow had embedded in them the value of research, of continuous enquiry, aiming to improve individual and overall patient care; it is the enthusiastic, unrelenting pursuit of progress that may well be based on an individual’s vision but can only be enabled through networking and collaboration; it is, in the case of some individuals, an even more personal interest and investment, that only they now know and treasure.

